# Comparison of knee articular cartilage thickness across sex and age groups in healthy adults

**DOI:** 10.1002/jeo2.70490

**Published:** 2025-10-30

**Authors:** Suwit Ariyachaikul, Sompong Sriburee, Nipaporn Thonglorm, Rungtiwa Kanthain, Amornthep Jankaew

**Affiliations:** ^1^ Department of Physical Therapy, Faculty of Associated Medical Sciences Chiang Mai University Chiang Mai Thailand; ^2^ Department of Radiologic Technology, Faculty of Associated Medical Sciences Chiang Mai University Chiang Mai Thailand

**Keywords:** age group, articular cartilage thickness, knee, sex, ultrasound

## Abstract

**Purposes:**

Cartilage thickness could be a significant biomarker for the early identification of knee osteoarthritis. However, reference data on cartilage thickness across various age groups are still lacking. Consequently, this study aims to establish reference values for cartilage thickness while examining differences related to sex and age groups.

**Methods:**

The study included 232 participants. Bilateral measurements of knee articular cartilage thickness were conducted using ultrasound (US) at three anatomical sites: the lateral femoral condyle, medial femoral condyle and intercondylar area. Three readings were taken per site by an experienced radiologist with expertise in musculoskeletal US. The mean cartilage thickness was calculated, and comparisons were performed between sexes using independent samples *t*‐tests and across various age groups (young, middle‐aged, older adults and retirement age) using one‐way analysis of variance.

**Results:**

Male participants demonstrated significantly higher cartilage thickness than females across all examined locations, except the middle area of the right knee. Notable differences among age groups were seen in the right lateral (*p* = 0.002) and medial (*p* = 0.019) compartments, along with the left medial compartment (*p* = 0.008). Analysis of the male subgroups indicated that those in the retirement age category exhibited greater cartilage thickness in the right lateral and medial compartments, as well as the left medial compartment, compared to other age groups.

**Conclusion:**

Males showed greater cartilage thickness than females across most knee areas. Additionally, age‐related variations in cartilage thickness were primarily observed in males, particularly in those of retirement age, while no significant age‐related differences were detected in females. These results underscore the importance of considering sex and age when evaluating knee cartilage health using US.

**Level of Evidence:**

Level III.

**Abbreviations**
BMI: body mass index
CI: confidence interval
CV%: coefficient of variation percentage
ICC: intra‐class correlation coefficient
MRI: magnetic resonance imaging
OA: osteoarthritis
SEM%: standard error of measurement percentage
SSE: sum of squares error
US: ultrasound

## INTRODUCTION

Osteoarthritis (OA) is a chronic disease that can affect all age groups, but it becomes increasingly prevalent as people age, contributing to a significant loss of disability‐adjusted life years in both female and male populations [9]. Knee OA, which affects approximately 22.9% of the elderly population [6], is characterised by joint degeneration and inflammation, in which the secretion of signalling molecules stimulates chondrocytes to generate matrix metalloproteinases, degradative enzymes involved in cartilage breakdown [15, 41]. Consequently, knee OA is now recognised as a chronic, multifactorial condition that develops due to an imbalance between anabolic and catabolic processes triggered by mechanical loading, impacting not only the articular cartilage but also other components of the knee joint [33]. Therefore, assessing these structures may play a crucial role in monitoring and the early detection of knee OA, potentially enhancing the effectiveness of exercise‐based prevention programmes.

Earlier research has demonstrated that assessing and monitoring changes in articular cartilage is beneficial for evaluating the effectiveness of health promotion programmes intended to prevent OA, along with treatment interventions aimed at alleviating the severity of joint destruction [11]. Several factors, including age, physical activity, obesity, biochemical markers [31, 35], gait mechanics and subchondral bone density, impact cartilage thickness [13]. Numerous studies consistently show that males possess notably greater knee cartilage thickness and volume compared to females across various knee areas [30]. Furthermore, it is acknowledged that the thickness of articular cartilage typically declines with age, particularly becoming noticeable in older adults. However, the rate and pattern of cartilage thinning can differ between sexes and knee regions, implying that both the formation of cartilage in early adulthood and its deterioration in later years play a role in age‐related variances in cartilage morphology [8]. This information highlights the necessity of assessing cartilage thickness across different sex and age demographics and indicates its potential in tracking the effectiveness of prevention and rehabilitation strategies aimed at mitigating joint destruction [4].

Radiography is a common method for assessing joint space narrowing in knee OA, but it has limitations in detecting cartilage and soft tissue degeneration, which can lead to underdiagnosis of early or asymptomatic cases [32]. Conversely, magnetic resonance imaging (MRI) is the gold standard for detailed structural evaluations, providing high accuracy and sensitivity for visualising the structures of the knee joint [20]. However, MRI is expensive and less accessible in routine clinical settings. High‐resolution ultrasound (US) has emerged as a promising non‐invasive alternative to address these limitations, effectively evaluating cartilage thickness and various joint pathologies [28, 32]. Evidence indicates that US measurements of cartilage thickness demonstrate moderate to high intra‐ and inter‐rater reliability and show a good correlation with MRI results (*r* = 0.67–0.82) [29, 36] and radiographic joint space narrowing (*r* ≈ 0.71) [27]. Although US may underestimate absolute cartilage thickness when compared to MRI, it is still a valuable tool for assessing cartilage health.

Assessing cartilage thickness is crucial for monitoring the health of articular cartilage in both healthy individuals and those with degenerative joint diseases [42]. However, there is a lack of evidence regarding reference values for articular cartilage thickness across various age groups when using US for measurement. Thus, this study aims to establish normative values for knee articular cartilage thickness in healthy adults aged 18–70 years. Furthermore, the research will investigate differences in knee cartilage thickness according to sex and age. It was hypothesised that cartilage thickness would differ significantly between sexes and across age groups, with males and younger individuals exhibiting greater thickness. By generating normative data and examining these relationships, the research aims to provide valuable insights into the factors that influence knee cartilage health in adults. The results are expected to improve the understanding of musculoskeletal health and guide future preventive measures to maintain joint integrity.

## METHODS

### Study design

A cross‐sectional design was used in this study. Crawford and Howell's [5] research indicates that an ideal sample size for determining normal reference values is about 50 subjects. This size facilitates the use of *t*‐tests or *z*‐tests for mean analysis, thanks to the improved normality of the distribution, which leads to narrower confidence intervals (CIs). Given that this study aims to assess the thickness of articular cartilage in adults, participants were divided into four age groups: young adulthood (18–24 years), middle adulthood (25–44 years), older adulthood (45–60 years) and retirement age (60–70 years) [39]. To ensure robust comparisons, the study maintained equal sample sizes across all age groups, with 50 participants in each, totalling 200 participants. Considering a 10% attrition rate, recruitment was adjusted to target 220 participants. The study was conducted in the laboratory setting of the university. Locations for the study were chosen using a purposive sampling method, followed by stratified random sampling based on the relevant age and sex groups. All participants were provided with detailed information regarding the testing procedures and gave written informed consent before participating. The research protocol received approval from the Research Ethics Committee of the Faculty of Associated Medical Sciences at Chiang Mai University, Thailand, in accordance with the principles outlined in the Declaration of Helsinki. The ethical approval reference number is AMSEC‐65EX‐040.

### Participants

A total of 232 healthy individuals of varying ages were enrolled in the present study. Participants had to be healthy and between 18 and 70 years old, regardless of gender. Exclusion criteria included individuals with knee OA by a physician based on x‐ray imaging in accordance with American College of Rheumatology criteria [1]; individuals whose US images revealed an irregular or non‐smooth cartilage rim, suggesting potential structural abnormalities; individuals with a history of musculoskeletal disorders or knee surgeries affecting mobility or balance; those with inflammatory joint diseases; individuals with visual impairments (either congenital or acquired), neurological or cardiovascular issues, congenital or acquired deformities of the lower limbs or spine, psychiatric disorders, or severe hearing loss; those who had been hospitalised in the past 3 months; individuals currently receiving chemotherapy or radiotherapy for cancer; participants with Stage 4 or 5 chronic kidney disease, and individuals with hormonal disorders. Participants were advised to refrain from sedatives or alcohol consumption for 24 h, and strenuous exercise for 48 h before the study. Table 1 presents the characteristics and baseline data for the total cohort, as well as data divided by sex.

**Table 1:** Participant characteristics.

	All (*n* = 232)^a^	Female (*n* = 132)^a^	Male (*n* = 100)^a^	*p* value
Age group (*n*, %)				0.710#
Young adulthood (18–24 years)	56 (24.1)	33 (25.0)	23 (23.0)	
Middle adulthood (25–44 years)	70 (30.2)	43 (32.6)	27 (27.0)	
Older adulthood (45–59 years)	47 (20.3)	25 (18.9)	22 (22.0)	
Retirement age (≥60 years)	59 (25.4)	31 (23.5)	28 (28.0)	
Height (cm)	162.1 ± 8.6	156.9 ± 6.3	169.1 ± 6.0	<0.001$
Weight (kg)^b^	61.0 (16.2)	54.0 (12.8)	67.5 (13.8)	<0.001$$
BMI (kg/m^2^)^b^	23.05 (5.76)	22.19 (5.77)	23.89 (4.67)	<0.001$
BMI group (*n*/%)				<0.01#
Underweight (<18.50)	25 (10.8)	21 (15.9)	4 (4.0)	
Healthy weight (18.50−22.99)	88 (37.9)	57 (43.2)	31 (31.0)	
Overweight (23.00–24.99)	41 (17.7)	16 (12.1)	25 (25.0)	
Obese I (25.00–29.99)	64 (27.6)	31 (23.5)	33 (33.0)	
Obese II (≥30.00)	14 (6.0)	7 (5.3)	7 (7.0)	

Abbreviation: BMI, body mass index.

^a^ Mean ± standard deviation, otherwise specified.

^b^ Non‐normal distribution or ordinal scale; median (interquartile range, IQR).

^$^ Independent *t*‐test.

^$$^ Mann–Whitney *U* test.

^#^ Fisher's exact test.

### Procedures

**Figure 1:**
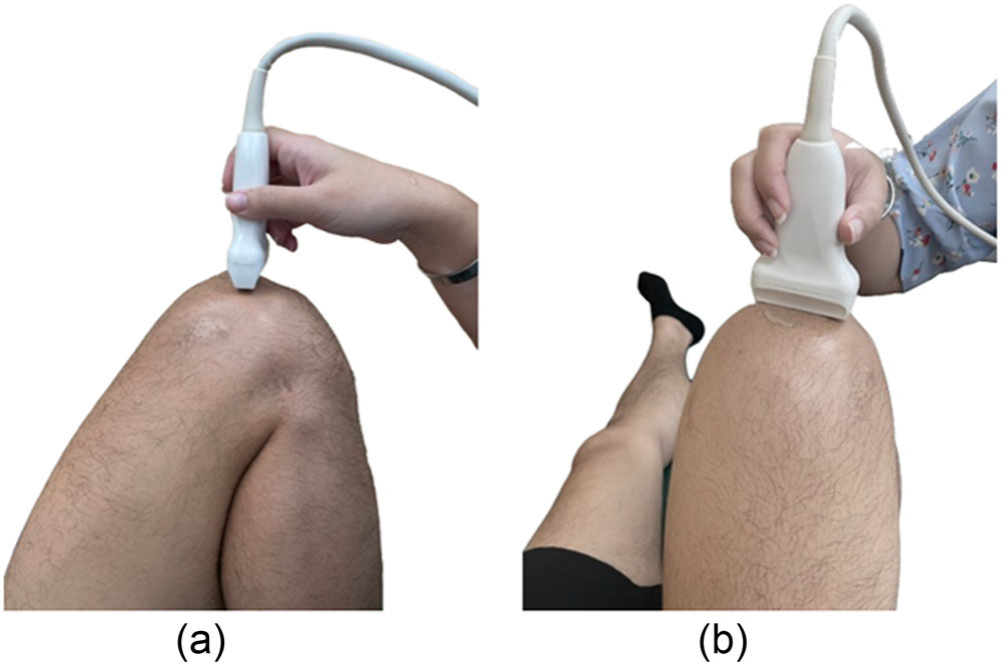
Participant positioning and ultrasound probe placement for knee assessment: (a) lateral view and (b) frontal view.

Data collection commenced with participants sharing their anthropometric information, including age and sex, followed by health‐related metrics such as weight, height and body mass index (BMI). Experienced radiologists assessed knee articular cartilage thickness using a 2D B‐mode US (CHISON ECO3 Expert Model US Diagnostic System) equipped with a linear probe set between 7 and 11 MHz. Participants lay supine on a treatment bed, with the tested knee positioned at 120 degrees of flexion and fully relaxed, as adjusted by a physical therapist. A goniometer was used to measure and confirm the knee flexion angle. The suprapatellar area was designated as the target for assessment. The US probe was positioned transversely over this area, ensuring perpendicular alignment to the femoral cartilage surface. Adjustment of the probe was necessary to achieve a clear image of the distal femoral cartilage, which showed a strong anechoic signal situated between the sharp bony cortex and the suprapatellar fat pad (Figure 1). Once the best image was captured, it was saved as a still image for subsequent analysis. Measurements were taken consecutively three times at three locations: the lateral femoral condyle, the intercondylar area and the medial femoral condyle by an experienced radiologist with six years of musculoskeletal US experience. Three images were acquired utilising consistent procedures, with the probe being removed from the knee following the acquisition of each image. This approach enabled the determination of the mean cartilage thickness at each site.

**Figure 2:**
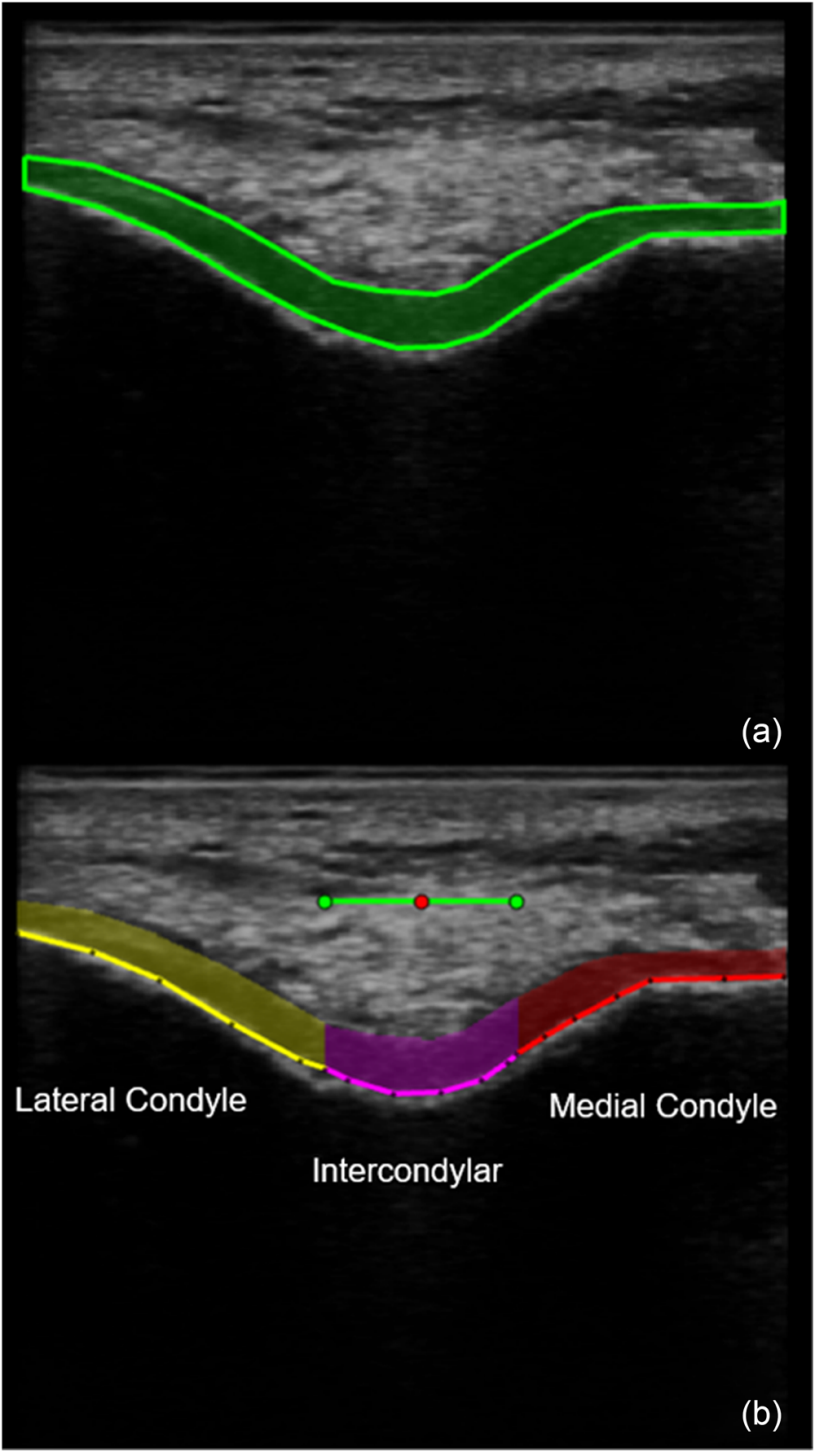
Measurement of articular cartilage thickness in the knee joint: manual segmentation of the overall cartilage region (a) and automated subdivision of the manually segmented cartilage into standardised cartilage regions—the medial condyle, intercondylar area and lateral condyle—using a custom program (b).

US image processing was conducted using a custom MATLAB program (R2020a, The MathWorks, Natick, MA, USA), which automatically executed multiple steps to calculate the average cartilage thickness. This analysis was conducted by a skilled radiologist specialising in US image interpretation, with six years of experience in musculoskeletal US. Initially, the analysts examined all images. They imported the images into the programme, manually locating and adjusting the segmentation to align with the bone shape. Subsequently, they identified the central point of the intercondylar notch on each image at its deepest point along the synovial‐cartilage boundary within standardised cartilage regions. The program then automatically divided the total cartilage cross‐sectional area into three standardised zones: medial, intercondylar (middle) and lateral. The intercondylar region was centred on the manually identified central point of the intercondylar notch, representing the middle 25% of cartilage width across the entire image [17]. The medial and lateral areas were defined as those medial and lateral to the intercondylar region, respectively. The intercondylar cartilage zone covered the central 25% of the image width, spanning 4.8 mm on either side of the notch's midpoint, ensuring sufficient coverage of the intercondylar notch while allowing the medial and lateral regions to include the slopes of the femoral condyles [17, 21]. The programme calculated the mean length of the cartilage–bone interface for each zone. In this study, cartilage thickness, defined as the cartilage cross‐sectional area divided by the length of the cartilage–bone interface, was assessed for the medial, intercondylar and lateral zones and averaged across three images (Figure 2).

### Statistical analysis

The data analysis was conducted using the SPSS software, version 30.0 (IBM Corp., Armonk, NY, USA). To evaluate data distribution for normality, the Shapiro–Wilk test was employed, particularly for samples with fewer than 50 observations. Results for normally distributed data were presented as mean and standard deviation, while non‐normally distributed data were reported as median and interquartile range. The intra‐rater reliability of US scans measuring knee articular cartilage thickness was assessed using intra‐class correlation coefficients (ICC_3,1_) with 95% CIs, based on a two‐way mixed‐effects model that assessed the consistency of agreement. Furthermore, the lower bound of the one‐sided 95% CI for ICC_3,1_ was computed, with values exceeding 0.70 being deemed acceptable [10, 34]. Absolute reliability was measured using the standard error of measurement percentage (SEM%) and the coefficient of variation percentage (CV%). The SEM% was calculated as the square root of the mean square error, with its 95% CI derived from the sum of squares error (SSE) and the chi‐square distribution, according to the ICC_3,1_ analysis of variance table [38]. An SEM% below 10% indicated minimal random error, regardless of measurement units [14]. The CV% and its 95% CI were derived using a logarithmic method [3], with values below 10% considered acceptable, indicating small variability within 10% of the mean [2].

## RESULTS

### Comparison of articular cartilage thickness of the knee between sexes

US imaging of knee articular cartilage showed acceptable reliability across all measurement sites for both female and male participants. Relative reliability, evaluated using the two‐way mixed‐effects ICC_3,1_, varied from 0.91 to 0.99, suggesting excellent consistency. All lower bounds of the one‐sided 95% CIs for ICC_3,1_ fell within acceptable limits. For absolute reliability, the SEM% ranged from 1.3% to 2.3%, with the CV% averaging between 1.25% and 2.27%.

In females, the average cartilage thickness of the right knee was 3.118 mm, while in males, it was 3.294 mm. For the left knee, the averages were 3.079 mm for females and 3.337 mm for males. A comparative analysis by sex indicated that males had significantly thicker cartilage than females at most anatomical locations. Notably, in the right knee, males exhibited significantly greater cartilage thickness in both the lateral and medial compartments compared to females (all *p* < 0.001). Conversely, in the left knee, males demonstrated significantly greater cartilage thickness across all assessed sites (all *p* < 0.001; Table 2).

**Table 2:** Comparison of articular cartilage thickness of the knee between sexes.

	All (*n* = 232)^a^	Female (*n* = 132)^a^	Male (*n* = 100)^a^	*p* value
Knee cartilage thickness (mm)				
Right, lateral	3.380 ± 0.385	3.288 ± 0.406	3.502 ± 0.320	<0.001$
Right, middle	2.880 ± 0.252	2.856 ± 0.243	2.911 ± 0.262	0.096$
Right, medial	3.320 ± 0.407	3.210 ± 0.381	3.468 ± 0.395	<0.001$
Left, lateral	3.364 ± 0.387	3.225 ± 0.353	3.548 ± 0.354	<0.001$
Left, middle	2.902 ± 0.294	2.839 ± 0.284	2.984 ± 0.288	<0.001$
Left, medial	3.305 ± 0.409	3.172 ± 0.362	3.479 ± 0.404	<0.001$

^a^ Mean ± standard deviation.

^$^ Independent *t*‐test.

### Comparison of articular cartilage thickness of the knee between age groups

Cartilage thickness showed significant variations across age groups in the right lateral (*F* = 4.924, *p* = 0.002) and medial (*F* = 3.393, *p* = 0.019) compartments, along with the left medial compartment of the knee (*F* = 4.061, *p* = 0.008). Post hoc analyses revealed that individuals in the retirement age group had significantly thicker cartilage than those in the middle adulthood group in both the right lateral (*p* < 0.001) and medial compartments (*p* < 0.05). Additionally, the retirement age group displayed significantly greater thickness than the young adulthood group in the right medial compartment (*p* < 0.05) and compared to the older adulthood group in the left medial compartment (*p* < 0.05). No statistically significant differences were found in the middle compartments of both knees overall (Table 3).

**Table 3:** Comparison of articular cartilage thickness of the knee between age groups.

	Gr.1 Young adulthood (*n* = 56)	Gr.2 Middle adulthood (*n* = 70)	Gr.3 Older adulthood (*n* = 47)	Gr.4 Retirement age (*n* = 59)	Sig. pair^$^
Knee cartilage thickness (mm)^a^					
Right, lateral	3.468 ± 0.420	3.271 ± 0.338	3.312 ± 0.374	3.481 ± 0.376	4 > 2,** 1 > 2*
Right, middle	2.855 ± 0.258	2.909 ± 0.266	2.878 ± 0.240	2.869 ± 0.243	NS
Right, medial	3.266 ± 0.391	3.273 ± 0.359	3.275 ± 0.404	3.464 ± 0.450	4 > (1,2)*
Left, lateral	3.434 ± 0.408	3.269 ± 0.391	3.331 ± 0.323	3.439 ± 0.391	NS
Left, middle	2.881 ± 0.329	2.952 ± 0.269	2.911 ± 0.270	2.854 ± 0.305	NS
Left, medial$	3.248 + 0.461	3.324 + 0.351	3.179 + 0.330	3.436 + 0.446	4 > 3*

^a^ Mean ± standard deviation.

* Statistically significant difference at *p* < 0.05.

** Statistically significant difference at *p* < 0.001.

^$^ All dependent variables except right medial thickness met the homogeneity of variance assumption, then one‐way analysis of variance (ANOVA) and Tukey honestly significant difference (HSD) post hoc test were used for multiple comparison. For right medial thickness, Kruskal–Wallis ANOVA and Bonferroni's correction were used for multiple comparisons. The number represents the groups that were compared.

When analysed by sex, the female population showed no significant variations in knee cartilage thickness across different age groups (Table 4). In contrast, the male population revealed statistically significant differences that aligned with the overall trend seen in the combined sample (right lateral: *F* = 3.627, *p* = 0.016; right medial: *F* = 4.206, *p* = 0.008; left medial: *F* = 4.614, *p* = 0.005). Specifically, males in the retirement age group exhibited the thickest articular cartilage, especially in the medial and lateral compartments of both knees. This group demonstrated significantly greater thickness than the older adulthood group in the right lateral and left medial compartments, and compared to the young and middle adulthood groups in the right medial compartment (all *p* < 0.05; Table 5).

**Table 4:** Comparison of knee articular cartilage thickness among age groups in females (*n* = 132).

	Gr.1 Young adulthood (*n* = 33)	Gr.2 Middle adulthood (*n* = 43)	Gr.3 Older adulthood (*n* = 25)	Gr.4 Retirement age (*n* = 31)	Sig. pair$
Knee cartilage thickness (mm)^a^					
Right, lateral	3.387 ± 0.444	3.182 ± 0.337	3.249 ± 0.435	3.361 ± 0.406	NS
Right, middle	2.856 ± 0.246	2.870 ± 0.262	2.835 ± 0.209	2.853 ± 0.249	NS
Right, medial	3.201 ± 0.375	3.212 ± 0.389	3.142 ± 0.327	3.271 ± 0.422	NS
Left, lateral	3.305 ± 0.358	3.162 ± 0.371	3.208 ± 0.315	3.242 ± 0.348	NS
Left, middle	2.826 ± 0.306	2.899 ± 0.251	2.878 ± 0.270	2.739 ± 0.299	NS
Left, medial	3.098 ± 0.425	3.201 ± 0.304	3.125 ± 0.320	3.250 ± 0.392	NS

^a^ Mean ± standard deviation.

^$^ One‐way analysis of variance (ANOVA) and post hoc Tukey honestly significant difference (HSD) for multiple comparisons were used.

**Table 5:** Comparison of knee articular cartilage thickness among age groups in males (*n* = 100).

	Gr.1 Young adulthood (*n* = 23)	Gr.2 Middle adulthood (*n* = 27)	Gr.3 Older adulthood (*n* = 22)	Gr.4 Retirement age (*n* = 28)	Sig. pair$
Knee cartilage thickness (mm)^a^					
Right, lateral	3.584 ± 0.361	3.412 ± 0.293	3.384 ± 0.283	3.614 ± 0.293	4 > 3*
Right, middle	2.855 ± 0.281	2.972 ± 0.264	2.927 ± 0.267	2.887 ± 0.238	NS
Right, medial	3.361 ± 0.403	3.370 ± 0.285	3.427 ± 0.437	3.678 ± 0.383	4 > (1,2)*
Left, lateral	3.619 ± 0.411	3.439 ± 0.367	3.471 ± 0.277	3.656 ± 0.316	NS
Left, middle	2.960 ± 0.351	3.037 ± 0.279	2.950 ± 0.271	2.981 ± 0.261	NS
Left, medial	3.464 ± 0.432	3.518 ± 0.339	3.241 ± 0.338	3.642 ± 0.415	4 > 3*

^a^ Mean ± standard deviation.

* Statistically significant difference at *p* < 0.05.

^$^ One‐way analysis of variance (ANOVA) and post‐hoc Tukey honestly significant difference (HSD) for multiple comparisons were used.

## DISCUSSION

This study established reference values for knee articular cartilage thickness in the middle, medial and lateral compartments across various age groups. It also compared cartilage thickness between genders and different age categories. The findings partially supported our hypothesis that males have thicker knee articular cartilage than females. However, significant differences in cartilage thickness were found across age groups, with the retirement age group showing notably thicker medial and lateral knee compartments compared to some younger groups. This age‐related pattern was primarily seen in males and was not present in females.

Measuring knee articular cartilage is crucial for diagnosing, monitoring and managing musculoskeletal disorders, such as OA, as it provides vital insights into cartilage health and disease progression [4]. Recent advancements in imaging techniques, particularly high‐resolution US, have enhanced the quantitative assessment of cartilage structure in both healthy individuals and those with medical conditions [28]. In this study, the average articular cartilage thickness in the knee, calculated across both knees and all measurement locations, was found to be 3.19 ± 0.24 mm. When analysed regionally for both sexes, the average thickness measured 3.31 ± 0.01 mm in the medial area, 2.89 ± 0.02 mm in the mid‐region and 3.37 ± 0.01 mm in the lateral area. Compared to prior studies of Asian populations, our results suggest a slightly higher cartilage thickness. For instance, research from Japan and South Korea has reported medial condyle thickness values ranging from 2.5 to 3 mm and lateral condyle values between 2.8 and 3.1 mm, which are lower than what we observed. Conversely, our findings align more closely with studies from European and North American populations. Specifically, research conducted in Italy and Spain has indicated femoral cartilage thicknesses of approximately 3.1 mm in the medial condyle and 3.2 mm in the lateral condyle, which align with our measurements [19, 26]. Similarly, US studies in the United States have shown medial femoral cartilage thicknesses ranging from 3.0 to 3.5 mm, further validating our results [23].

There are notable differences in cartilage thickness in the knee joint between males and females. This study reveals that males generally have significantly thicker articular cartilage than females across most anatomical sites of the knee, especially in the lateral and medial compartments of both knees. These results align with recent findings highlighting sex‐specific differences in knee cartilage structure and volume. For instance, Di Martino et al. [7] conducted a systematic review of gender differences in hip and knee cartilage composition. They noted that males consistently exhibit greater cartilage volume and thickness than females from infancy to adulthood, with these differences becoming more pronounced after menopause due to hormonal and compositional changes in the cartilage. Biomechanically, larger joint sizes and bone dimensions in males likely lead to thicker cartilage to support higher load‐bearing demands [8, 37]. Additionally, hormonal factors, such as oestrogen, affect cartilage metabolism and may account for the thinner cartilage and heightened degeneration risk found in females [18]. Furthermore, longitudinal imaging studies indicate that women experience faster cartilage loss and a quicker progression of cartilage defects compared to men, even before the clinical onset of OA, suggesting an inherent vulnerability of cartilage related to sex [16]. Understanding these sex‐specific differences may inform the development of targeted strategies for the prevention and management of knee OA based on gender.

Knee articular cartilage thickness typically declines with age, primarily due to degenerative changes linked to ageing [37]. However, analysis across four age groups showed that the retirement age group had greater cartilage thickness than the young, middle‐aged and older adult groups, especially in the medial and lateral compartments. Additionally, we conducted subgroup analyses to assess knee cartilage thickness across various age groups within each sex. In the female group, no notable differences in cartilage thickness were found among the age groups. Conversely, the male group revealed significant changes in cartilage thickness across age groups, aligning with the overall trend observed when combining both sexes. Previous studies have indicated that age‐related changes in cartilage thickness are not consistent across all knee regions; some areas, particularly the medial and lateral compartments, may experience relative thickening or less thinning in specific age groups due to variations in mechanical loading or biological responses [37]. In our context, this can be attributed to the fact that reaching retirement age often corresponds with shifts in physical activity patterns, which may foster more regular moderate exercise that supports joint health without causing excessive wear. This could potentially help maintain or even enhance cartilage thickness compared to younger adults by stimulating chondrocytes to improve the synthesis of extracellular matrix components. Previous research indicates that moderate mechanical stress, associated with moderate exercise intensity, encourages chondrocytes to increase the production of extracellular matrix components, such as proteoglycans and collagen. This process enhances cartilage thickness and improves tissue quality, further supported by the upregulation of growth factors, which facilitate chondrocyte biosynthesis and cartilage matrix production [24, 40]. Therefore, regular physical activity and exercise among retirees may serve as a protective, chondroprotective stimulus that helps maintain or increase cartilage thickness in this demographic, in contrast to younger groups with more inconsistent activity levels or occupational stresses [25]. Additionally, the observed increase in cartilage thickness among retirees might represent early degenerative changes, including cartilage swelling and higher water content, which occur before matrix breakdown [22]. Hence, the differences observed in cartilage thickness among retirees compared to other groups likely stem from a mix of region‐specific cartilage responses, mechanical loading effects, hormonal influences and lifestyle changes, rather than merely reflecting a linear decline with age.

To the best of the author's knowledge, this study is one of the first to analyse knee articular cartilage thickness across genders and a broad age spectrum, from young adults to retirees, using high‐resolution US technology, which is reliable, cost‐effective and safe for patients [28, 32]. Our findings offer normative US cartilage thickness values for the general population that align with international figures reported in earlier studies. Clinicians can utilise our data as a reference for evaluating and monitoring cartilage health in both healthy and pathological individuals, promoting early detection of cartilage degeneration. Additionally, recognising age‐ and sex‐specific cartilage traits may inform the creation of targeted interventions to maintain joint integrity and hinder OA progression.

The current study has several limitations that need to be addressed. First, the exclusive use of US imaging, although reliable for assessing cartilage thickness in the anterior femoral condyles and trochlea, does not allow comprehensive visualisation of the posterior condylar regions, as MRI does. The absence of MRI assessments therefore limits the comprehensive evaluation of cartilage health across all knee compartments. Second, since participants were recruited from the general population without stratification by profession or activity level, potential differences in joint loading between individuals or between sexes could not be assessed. Future studies should consider collecting and analysing these variables to better understand their impact on cartilage health. Third, being a cross‐sectional study, it restricts the capacity to observe changes in cartilage thickness over time. To capture long‐term variations in knee articular cartilage thickness, future longitudinal studies are essential. Such research could shed light on the progression or maintenance of cartilage health. Finally, numerous internal and external factors, such as BMI, physical activity, metabolic health and biomechanical loading, can affect cartilage thickness [12]. Future studies should explore the relationships between these factors and cartilage thickness to gain a deeper understanding of the mechanisms influencing cartilage morphology and to identify modifiable risk factors for either cartilage degeneration or preservation.

## CONCLUSION

This study examined the thickness of knee articular cartilage and provided normative values in healthy adults across a wide age range. The findings revealed significant differences related to sex, with males showing greater cartilage thickness than females. Furthermore, individuals in the retirement age group had thicker cartilage compared to younger cohorts, especially in the medial and lateral compartments of the knee. The normative US cartilage thickness values established in this research offer a valuable reference for both clinicians and researchers. Clinicians can use this data to enhance the assessment and monitoring of cartilage health in various adult populations, supporting early detection of degenerative changes and guiding personalised management strategies.

## AUTHOR CONTRIBUTIONS

*Conceptualisation*: Suwit Ariyachaikul and Sompong Sriburee. *Methodology*: Suwit Ariyachaikul and Sompong Sriburee. *Formal analysis and investigation*: Suwit Ariyachaikul, Sompong Sriburee, Nipaporn Thonglorm and Rungtiwa Kanthain. *Writing—original draft preparation*: Suwit Ariyachaikul and Amornthep Jankaew. *Writing—review and editing*: Suwit Ariyachaikul and Amornthep Jankaew. *Funding acquisition*: Suwit Ariyachaikul. *Supervision*: Suwit Ariyachaikul.

## CONFLICT OF INTEREST STATEMENT

The authors declare no conflicts of interest.

## ETHICS STATEMENT

This study was approved by the Research Ethics Committee of the Faculty of Associated Medical Sciences, Chiang Mai University, Chiang Mai, Thailand. The ethical approval number is AMSEC‐65EX‐040. All participants provided informed consent before their participation in the study.

## PATIENT CONSENT STATEMENT

Not applicable.

## PERMISSION TO REPRODUCE MATERIAL FROM OTHER SOURCES

Not applicable.

## FOR CLINICAL TRIALS

Not applicable.

## Data Availability

The data that support the findings of this study are available from the corresponding author upon reasonable request.

## References

[1] Altman R , Asch E , Bloch D , Bole G , Borenstein D , Brandt K , et al. Development of criteria for the classification and reporting of osteoarthritis. Classification of osteoarthritis of the knee. Arthritis Rheum. 1986;29:1039–1049. 3741515 10.1002/art.1780290816

[2] Atkinson G , Nevill AM . Statistical methods for assessing measurement error (reliability) in variables relevant to sports medicine. Sports Med. 1998;26:217–238. 9820922 10.2165/00007256-199826040-00002

[3] Bland JM , Altman DG . Statistics notes: measurement error proportional to the mean. BMJ. 1996;313:106. 8688716 10.1136/bmj.313.7049.106 PMC2351517

[4] Brody LT . Knee osteoarthritis: clinical connections to articular cartilage structure and function. Phys Ther Sport. 2015;16:301–316. 25783021 10.1016/j.ptsp.2014.12.001

[5] Crawford JR , Howell DC . Comparing an individual's test score against norms derived from small samples. Clin Neuropsychol. 1998;12:482–486.

[6] Cui A , Li H , Wang D , Zhong J , Chen Y , Lu H . Global, regional prevalence, incidence and risk factors of knee osteoarthritis in population‐based studies. EClinicalMedicine. 2020;29–30:100587. 10.1016/j.eclinm.2020.100587 PMC7704420 34505846

[7] Di Martino A , Barile F , D'Agostino C , Castafaro V , Cerasoli T , Mora P , et al. Are there gender‐specific differences in hip and knee cartilage composition and degeneration? A systematic literature review. Eur J Orthop Surg Traumatol. 2024;34:1901–1910. 38456943 10.1007/s00590-024-03871-4 PMC11101511

[8] Ding C . Sex differences in knee cartilage volume in adults: role of body and bone size, age and physical activity. Rheumatology. 2003;42:1317–1323. 12810930 10.1093/rheumatology/keg374

[9] Ding Y , Liu X , Chen C , Yin C , Sun X . Global, regional, and national trends in osteoarthritis disability‐adjusted life years (DALYs) from 1990 to 2019: a comprehensive analysis of the global burden of disease study. Public Health. 2024;226:261–272. 38134839 10.1016/j.puhe.2023.10.030

[10] Dobson F , Hinman RS , Hall M , Marshall CJ , Sayer T , Anderson C , et al. Reliability and measurement error of the Osteoarthritis Research Society International (OARSI) recommended performance‐based tests of physical function in people with hip and knee osteoarthritis. Osteoarthritis Cartilage. 2017;25:1792–1796. 28647467 10.1016/j.joca.2017.06.006

[11] Eckstein F , Collins JE , Nevitt MC , Lynch JA , Kraus VB , Katz JN , et al. Brief report: cartilage thickness change as an imaging biomarker of knee osteoarthritis progression: data from the foundation for the National Institutes of Health Osteoarthritis Biomarkers Consortium. Arthritis Rheum. 2015;67:3184–3189. 10.1002/art.39324 PMC5495918 26316262

[12] Eckstein F , Maschek S , Culvenor A , Sharma L , Roemer FW , Duda GN , et al. Which risk factors determine cartilage thickness and composition change in radiographically normal knees?—Data from the Osteoarthritis Initiative. Osteoarthritis Cartilage Open. 2023;5:100365. 37207279 10.1016/j.ocarto.2023.100365 PMC10188628

[13] Edd SN , Omoumi P , Andriacchi TP , Jolles BM , Favre J . Modeling knee osteoarthritis pathophysiology using an integrated joint system (IJS): a systematic review of relationships among cartilage thickness, gait mechanics, and subchondral bone mineral density. Osteoarthritis Cartilage. 2018;26:1425–1437. 30056214 10.1016/j.joca.2018.06.017

[14] Goldberg A , Casby A , Wasielewski M . Minimum detectable change for single‐leg‐stance‐time in older adults. Gait Posture. 2011;33:737–739. 21444208 10.1016/j.gaitpost.2011.02.020

[15] Grässel S , Zaucke F , Madry H . Osteoarthritis: novel molecular mechanisms increase our understanding of the disease pathology. J Clin Med. 2021;10:1938. 33946429 10.3390/jcm10091938 PMC8125020

[16] Hanna FS , Teichtahl AJ , Wluka AE , Wang Y , Urquhart DM , English DR , et al. Women have increased rates of cartilage loss and progression of cartilage defects at the knee than men: a gender study of adults without clinical knee osteoarthritis. Menopause. 2009;16:666–670. 19598333 10.1097/gme.0b013e318198e30e

[17] Harkey MS , Little E , Thompson M , Zhang M , Driban JB , Salzler MJ . Femoral cartilage ultrasound echo intensity associates with arthroscopic cartilage damage. Ultrasound Med Biol. 2021;47:43–50. 33082054 10.1016/j.ultrasmedbio.2020.09.015 PMC7568485

[18] Hernandez PA , Bradford JC , Brahmachary P , Ulman S , Robinson JL , June RK , et al. Unraveling sex‐specific risks of knee osteoarthritis before menopause: do sex differences start early in life? Osteoarthritis Cartilage. 2024;32:1032–1044. 38703811 10.1016/j.joca.2024.04.015 PMC12312443

[19] Herrera H GA , Llinás PJ , Flórez L , Blanco Montes C , Vernaza Obando D , Díaz Solorzano A , et al. Ultrasound measurement of femoral cartilage thickness in the knee of healthy young university students. Rev Esp Cir OrtopTraumatol (Engl Ed). 2020;64:244–250. 10.1016/j.recot.2020.04.001 32499158

[20] Lee LS , Chan PK , Fung WC , Chan VWK , Yan CH , Chiu KY . Imaging of knee osteoarthritis: a review of current evidence and clinical guidelines. Musculoskeletal Care. 2021;19:363–374. 33387447 10.1002/msc.1536

[21] Lisee C , McGrath ML , Kuenze C , Zhang M , Salzler M , Driban JB , et al. Reliability of a novel semiautomated ultrasound segmentation technique for assessing average regional femoral articular cartilage thickness. J Sport Rehabil. 2020;29:1042–1046. 32473587 10.1123/jsr.2019-0476

[22] Loeser RF , Collins JA , Diekman BO . Ageing and the pathogenesis of osteoarthritis. Nat Rev Rheumatol. 2016;12:412–420. 27192932 10.1038/nrrheum.2016.65 PMC4938009

[23] Lunser MK , Hurdle MF , Taylor WC , et al. Ultrasound measurement of femoral articular cartilage thickness before and after marathon running. Cureus. 2024;16:e52870. 38406107 10.7759/cureus.52870 PMC10894013

[24] Mauck RL , Yuan X , Tuan RS . Chondrogenic differentiation and functional maturation of bovine mesenchymal stem cells in long‐term agarose culture. Osteoarthritis Cartilage. 2006;14:179–189. 16257243 10.1016/j.joca.2005.09.002

[25] Mithoefer K , Hambly K , Logerstedt D , Ricci M , Silvers H , Villa SD . Current concepts for rehabilitation and return to sport after knee articular cartilage repair in the athlete. J Orthop Sports Phys Ther. 2012;42:254–273. 22383103 10.2519/jospt.2012.3665

[26] Naredo E , Acebes C , Möller I , Canillas F , de Agustín JJ , de Miguel E , et al. Ultrasound validity in the measurement of knee cartilage thickness. Ann Rheum Dis. 2009;68:1322–1327. 18684742 10.1136/ard.2008.090738

[27] Okano T , Filippucci E , Di Carlo M , Draghessi A , Carotti M , Salaffi F , et al. Ultrasonographic evaluation of joint damage in knee osteoarthritis: feature‐specific comparisons with conventional radiography. Rheumatology. 2016;55:2040–2049. 27558583 10.1093/rheumatology/kew304

[28] Okano T , Mamoto K , Di Carlo M , Salaffi F . Clinical utility and potential of ultrasound in osteoarthritis. Radiol Med (Torino). 2019;124(11):1101–1111. 30828773 10.1007/s11547-019-01013-z

[29] Østergaard M , Court‐Payen M , Gideon P , Wieslander S , Cortsen M , Lorenzen I , et al. Ultrasonography in arthritis of the knee: a comparison with MR imaging. Acta Radiol. 1995;36:19–26. 7833164

[30] Otterness IG , Eckstein F . Women have thinner cartilage and smaller joint surfaces than men after adjustment for body height and weight. Osteoarthritis Cartilage. 2007;15:666–672. 17321168 10.1016/j.joca.2006.12.003

[31] Pamukoff DN , Vakula MN , Holmes SC , Shumski EJ , Garcia SA . Body mass index moderates the association between gait kinetics, body composition, and femoral knee cartilage characteristics. J Orthop Res. 2020;38:2685–2695. 32162713 10.1002/jor.24655

[32] Piccolo CL , Mallio CA , Vaccarino F , Grasso RF , Zobel BB . Imaging of knee osteoarthritis: a review of multimodal diagnostic approach. Quant Imaging Med Surg. 2023;13:7582–7595. 37969633 10.21037/qims-22-1392 PMC10644136

[33] Primorac D , Molnar V , Rod E , Jeleč Ž , Čukelj F , Matišić V , et al. Knee osteoarthritis: a review of pathogenesis and state‐of‐the‐art non‐operative therapeutic considerations. Genes. 2020;11:854. 32722615 10.3390/genes11080854 PMC7464436

[34] Prinsen CAC , Vohra S , Rose MR , Boers M , Tugwell P , Clarke M , et al. How to select outcome measurement instruments for outcomes included in a “Core Outcome Set”—a practical guideline. Trials. 2016;17:449. 27618914 10.1186/s13063-016-1555-2 PMC5020549

[35] Roberts HM , Griffith‐McGeever CL , Owen JA , Angell L , Moore JP , Thom JM . An exploratory study to investigate the association between age, physical activity, femoral trochlear cartilage thickness and biomarkers of tissue metabolism in adult males. Eur J Appl Physiol. 2021;121:1871–1880. 33713200 10.1007/s00421-021-04655-y PMC8192398

[36] Schmitz RJ , Wang HM , Polprasert DR , Kraft RA , Pietrosimone BG . Evaluation of knee cartilage thickness: a comparison between ultrasound and magnetic resonance imaging methods. Knee. 2017;24:217–223. 27914723 10.1016/j.knee.2016.10.004

[37] Si L , Xuan K , Zhong J , Huo J , Xing Y , Geng J , et al. Knee cartilage thickness differs alongside ages: a 3‐T magnetic resonance research upon 2,481 subjects via deep learning. Front Med. 2020;7:600049. 10.3389/fmed.2020.600049 PMC7900571 33634142

[38] Stratford PW , Goldsmith CH . Use of the standard error as a reliability index of interest: an applied example using elbow flexor strength data. Phys Ther. 1997;77:745–750. 9225846 10.1093/ptj/77.7.745

[39] United Nations, Department of International Economic and Social Affairs, Statistical Office . 1982. Provisional guidelines on standard international age classifications. Statistical Papers Series M No. 74.

[40] Vincent TL , Wann AKT . Mechanoadaptation: articular cartilage through thick and thin. J Physiol. 2019;597:1271–1281. 29917242 10.1113/JP275451 PMC6395418

[41] Yao Q , Wu X , Tao C , Gong W , Chen M , Qu M , et al. Osteoarthritis: pathogenic signaling pathways and therapeutic targets. Signal Transduct Target Ther. 2023;8:56. 36737426 10.1038/s41392-023-01330-w PMC9898571

[42] Zhang H , Ning E , Lu L , Zhou J , Shao Z , Yang X , et al. Research progress of ultrasound in accurate evaluation of cartilage injury in osteoarthritis. Front Endocrinol. 2024;15:1420049. 10.3389/fendo.2024.1420049 PMC11358554 39211448

